# Separate and Unequal: Residential Segregation and Estimated Cancer Risks Associated with Ambient Air Toxics in U.S. Metropolitan Areas

**DOI:** 10.1289/ehp.8500

**Published:** 2005-10-19

**Authors:** Rachel Morello-Frosch, Bill M. Jesdale

**Affiliations:** 1 Department of Community Health, School of Medicine, and; 2 Center for Environmental Studies, Brown University, Providence, Rhode Island, USA

**Keywords:** air toxics, cancer risk, environmental justice, health disparity, racial disparity, segregation

## Abstract

This study examines links between racial residential segregation and estimated ambient air toxics exposures and their associated cancer risks using modeled concentration estimates from the U.S. Environmental Protection Agency’s National Air Toxics Assessment. We combined pollutant concentration estimates with potencies to calculate cancer risks by census tract for 309 metropolitan areas in the United States. This information was combined with socioeconomic status (SES) measures from the 1990 Census. Estimated cancer risks associated with ambient air toxics were highest in tracts located in metropolitan areas that were highly segregated. Disparities between racial/ethnic groups were also wider in more segregated metropolitan areas. Multivariate modeling showed that, after controlling for tract-level SES measures, increasing segregation amplified the cancer risks associated with ambient air toxics for all racial groups combined [highly segregated areas: relative cancer risk (RCR) = 1.04; 95% confidence interval (CI), 1.01–107; extremely segregated areas: RCR = 1.32; 95% CI, 1.28–1.36]. This segregation effect was strongest for Hispanics (highly segregated areas: RCR = 1.09; 95% CI, 1.01–1.17; extremely segregated areas: RCR = 1.74; 95% CI, 1.61–1.88) and weaker among whites (highly segregated areas: RCR = 1.04; 95% CI, 1.01–1.08; extremely segregated areas: RCR = 1.28; 95% CI, 1.24–1.33), African Americans (highly segregated areas: RCR = 1.09; 95% CI, 0.98–1.21; extremely segregated areas: RCR = 1.38; 95% CI, 1.24–1.53), and Asians (highly segregated areas: RCR = 1.10; 95% CI, 0.97–1.24; extremely segregated areas: RCR = 1.32; 95% CI, 1.16–1.51). Results suggest that disparities associated with ambient air toxics are affected by segregation and that these exposures may have health significance for populations across racial lines.

Nearly 80% of the approximately 280 million people living in the United States reside in metropolitan areas (U.S. Bureau of the Census 2004). Environmental health researchers and public health practitioners have recently begun to focus on the links between the urban built environment, social inequality, and community health and well-being (Frumkin 2002, 2003; Jackson 2002; Northridge et al. 2003). Despite the proliferation of research on this issue, there is a lack of scientific consensus about what it is about neighborhood and other area-level variables that affect health. Neighborhood-level factors affect individual health by influencing access to quality foods, especially fresh fruits and vegetables and affordable supermarkets, and access to crucial services, such as health care, parks, and open space (Diez-Roux 2003; Morland et al. 2002; Transportation and Land Use Coalition 2002). Other key neighborhood factors that affect health include the social environment (social capital, cohesion, and crime rates) (Kawachi and Berkman 2003; Wallace and Wallace 1998; Wallace 1988) and the physical environment (traffic density, housing quality, and abandoned properties) (Reynolds et al. 2002; Shenassa et al. 2004; Wallace 1990).

Environmental health researchers, sociologists, policy makers, and advocates concerned about environmental justice have argued that residents of color who are concentrated in neighborhoods with high levels of poverty are also disproportionately exposed to physical environments that adversely affect their health and well-being. Research on race and class differences in exposures to toxics varies widely, and although by no means unequivocal, much of the evidence suggests a pattern of disproportionate exposures to toxics and associated health risks among communities of color and the poor, with racial differences often persisting across economic strata (Burke 1993; Morello-Frosch et al. 2001, 2002a, 2002b; Pastor et al. 2001; Perlin et al. 2001; Sadd et al. 1999). Such evidence has important implications for policy making, but few studies elucidate links between social inequality and residential segregation with exposures to environmental hazards (Morello-Frosch 2002; Morello-Frosch et al. 2001).

Wide-ranging and complex political and socioeconomic forces, coupled with patterns of industrialization and development, have segregated people of color, particularly African Americans, into neighborhoods with some of the highest indices of urban poverty and deprivation (Peet 1984; Schultz et al. 2002; Walker 1985; Williams and Collins 2001, 2004). Indeed, uneven industrial development, real estate speculation, discrimination in government and private financing, workplace discrimination, and exclusionary zoning have led to systemic racial segregation among diverse communities with important implications for community health and individual well-being (Logan 1978; Logan and Molotch 1987; Morello-Frosch 2002; Sinton 1997; Wilson 1996). Studies connecting residential segregation to health outcomes and health disparities represent a relatively new direction of research. Much of this work has focused on the health impacts of residential segregation on African Americans (LaVeist 1989, 1992, 1993; Polednak 1991, 1993, 1996a, 1996b, 1997). Results of this research generally show that residential segregation is associated with elevated risks of adult and infant mortality (Collins and Williams 1999; LaVeist 1989, 1992, 1993; Polednak 1991, 1993, 1996a, 1996b, 1997; Williams and Collins 2001) and tuberculosis (Acevedo-Garcia 2001).

Although elements for understanding the relationship between residential segregation and community environmental health can be found separately in the literature of both sociology and environmental justice, only one previous investigation has attempted to combine these two lines of inquiry to analyze the relationship between outdoor air pollution exposure and segregation (Lopez 2002). Some researchers have recently argued that residential segregation is a crucial place to start for understanding the origins and persistence of environmental health disparities (Gee and Payne-Sturges 2004; Lopez 2002; Morello-Frosch 2002; Morello-Frosch et al. 2001; Pulido 1994, 2000; Pulido et al. 1996). Gee and Payne-Sturges (2004) propose a conceptual framework for understanding how race-based segregation may lead to a disproportionate burden of cumulative exposures to potential environmental hazards among certain communities while enhancing their vulnerability or susceptibility to the toxic effects of exposures due to individual and area-level stressors, and lack of neighborhood resources. In this study we seek to operationalize parts of this conceptual framework by examining links between racial residential segregation and estimated cancer risks associated with modeled ambient air toxics exposures. Recent analysis of modeled national estimates suggests that ambient concentrations of hazardous air pollutants (HAPs) exceed benchmark risk levels for cancer and noncancer end points in many areas of the country (Apelberg et al. 2005; Morello-Frosch et al. 2000; Woodruff et al. 1998). Follow-up studies on air quality as well as stationary and mobile sources of air pollution have found a disproportionate burden of exposures and associated cancer and noncancer health risks for communities of color and poor residents. These studies have examined transportation corridors with high traffic density (Gunier et al. 2003), location of Toxics Release Inventory (TRI) and other treatment, storage, and disposal facilities (Morello-Frosch et al. 2002a; Pastor et al. 2001, 2002; Perlin et al. 1999, 2001), and modeled estimates of air toxics from the U.S. Environmental Protection Agency (EPA) Cumulative Exposure Project (CEP) and National Air Toxics Assessment (NATA) (Lopez 2002; Morello-Frosch et al. 2002a, 2002b; Pastor et al. 2002, 2004). For this study, we assessed whether racial and economic disparities in estimated cancer risk associated with air toxics are modified by levels of residential segregation in U.S. metropolitan areas.

## Materials and Methods

To analyze the relationship between pollution and health risk burdens with race-based residential segregation, we obtained modeled ambient air toxics concentration estimates from the U.S. EPA’s NATA and combined these data with cancer potency information. We then integrated these cancer risk estimates with socioeconomic and demographic information derived from the 1990 U.S. Census (U.S. Census Bureau 1991, 1993) for all tracts within 309 metropolitan areas in the continental United States. All data linking, data management, and statistical analysis were performed using SAS (version 8.2; SAS Institute Inc., Cary, NC).

### Modeled estimates of outdoor air toxics concentrations.

The U.S. EPA’s most recent publicly accessible national-scale air toxics assessment was conducted for 1996 and estimates the annual average concentration for a subset of the 188 HAPs listed in section 112 of the 1990 Clean Air Act Amendments (33 pollutants, including diesel particulate matter). The methods used to generate census-tract–level estimates of risk are described in detail by the U.S. EPA and others (Rosenbaum et al. 1999; U.S. EPA 2005a). Using an algorithm based on the Assessment System for Population Exposure Nationwide (ASPEN) model, NATA generates concentration estimates using a Gaussian dispersion modeling approach that accounts for meteorologic conditions, wind speed, and atmospheric chemistry, including processes such as reactive decay, secondary pollutant formation, and deposition. NATA then applies the model algorithm to the U.S. EPA’s National Toxics Inventory, which is compiled using five primary information sources: state and local toxic air pollutant inventories, existing databases related to the U.S. EPA’s air toxics regulatory program, the U.S. EPA’s TRI database, estimates using mobile source methodology (developed by the U.S. EPA’s Office of Transportation and Air Quality), and emission estimates generated from emission factors and activity data (U.S. EPA 2005a).

The model then allocates air toxics concentration estimates in statewide grids that can be used to create data surfaces and for interpolation and allocation to census tracts (U.S. EPA 2005a). The model estimates long-term HAP concentrations attributable to anthropogenic sources within 50 km of each census tract centroid. Each pollutant concentration is a spatial average that approximates the population-weighted average of outdoor HAP concentrations experienced within a census tract over the course of a year. There are > 60,000 census tracts in the continental United States, with each averaging between 4,000 and 5,000 residents. Specifics of the model are discussed elsewhere (Rosenbaum et al. 1999; U.S. EPA 2005a). We assessed air toxics concentrations for stationary emissions sources, which include point-source emissions (from facilities required to report emissions to the TRI, including large chemical manufacturers, refineries, and electrical power plants) and smaller area sources (including dry cleaners, auto body shops, and chrome plating facilities); and for mobile emissions sources, which include on-road vehicles (e.g., trucks and cars) and nonroad sources (e.g., airplanes, trains, construction equipment, and farm equipment) (U.S. EPA 2005a). Estimated outdoor concentrations also included a background portion attributable to long-range transport, resuspension of historical emissions, and natural sources derived from measurements taken at clean air locations remote from known emissions sources. These values were treated as a constant across all census tracts and added to the modeled concentration estimates from mobile and stationary emissions sources.

### Assessment of cancer risks.

We combined modeled HAP concentration estimates with cancer potency information to estimate the distribution of cumulative cancer health risks in accordance with California’s “hot spots” guidelines [Office of Environmental Health Hazard Assessment (OEHHA) 2003]. The guidelines provide procedures for use in the preparation of cancer and noncancer health risk assessments required under California’s Air Toxics “Hot Spots” Information and Assessment Act (1987). This law established a statewide program for the inventory of air toxics emissions from individual facilities as well as requirements for risk assessment and public notification of potential health risk (OEHHA 2003).

We assessed cancer risks using inhalation unit risk (IUR) estimates in micrograms per cubic meter for each carcinogenic compound. Inhalation unit risk estimates are defined as the individual lifetime excess risk due to a chronic lifetime exposure to one unit of pollutant concentration (U.S. EPA 2003). Potency estimates generally assume nonthreshold, low-dose linearity unless there is compelling evidence to the contrary, and are derived from occupational or animal studies. The unit risk calculated from occupational studies is based on a maximum-likelihood estimate of the dose–response data. Potencies derived from animal data represent a 95% upper bound estimate of the probability of contracting cancer.

The U.S. EPA, the California Environmental Protection Agency (Cal-EPA), and the International Agency for Research on Cancer (IARC) identify carcinogens based on the sci-entific weight of evidence for carcinogenicity, which is derived from human and animal data. The weight-of-evidence descriptors for carcinogenicity used by various agencies vary somewhat, and the U.S. EPA is in the process of revising their cancer risk assessment guidelines (U.S. EPA 2003), but the categories used are similar. Currently, the U.S. EPA is proposing to classify potential carcinogens based on the following weight-of-evidence categories: *a*) carcinogenic to humans, *b*) likely to be carcinogenic to humans, *c*) suggestive evidence of carcinogenic potential, *d*) inadequate information to assess carcinogenic potential, *e*) not likely to be carcinogenic to humans. Air toxics classified in any of the first three descriptor categories were evaluated in this analysis (U.S. EPA 2003). We also used the California OEHHA (2002) IUR estimate for diesel particulates to calculate an estimated lifetime cancer risk for diesel particulates. Although the U.S. EPA does not have an IUR for diesel, Cal-EPA has derived a potency estimate for this mixture of compounds and has classified it as a carcinogen under Proposition 65 (OEHHA 2005). Similarly, IARC has classi-fied diesel particulates as a probable carcinogen (IARC 2005).

Estimated cancer risks for each pollutant in each census tract were derived with the following formula:


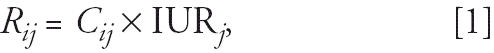


where *R_ij_* is the estimate of individual lifetime cancer risk from pollutant *j* in census tract *i*, *C_ij_* is the concentration of HAP *j* in micrograms per cubic meter in census tract *i*, and IUR is the IUR estimate for pollutant *j* in micrograms per cubic meter. The cancer risks of different air toxics were assumed to be additive and were summed together in each census tract to estimate a total individual lifetime cancer risk in each tract. To roughly estimate the number of cancer cases from lifetime exposures, we multiplied the total cancer risk in each census tract by the total tract population.

### 1990 census data.

The tract-level health risk data were matched with area level socioeconomic and demographic information from the 1990 Census (summary tapes file 1 and 3; U.S. Census Bureau 1991, 1993). These data were used to derive the following variables used in our analysis.

#### Segregation.

Massey and Denton have identified several conceptual dimensions of segregation, all of which were conceived with a particular context in mind: that of urban segregation of blacks from whites in the United States (Massey and Denton 1988, 1989; Massey et al. 1996; U.S. Bureau of the Census 2004). These concepts and measures have been expanded to consider the segregation of Hispanic-American and Asian-American populations from whites (Massey 2004; Massey and Fong 1990). To maximize congruence with the theory and development of the segregation indices, we have also constrained our analysis to metropolitan areas of the United States.

Of the various conceptual dimensions of segregation, evenness as measured by the dissimilarity index has most often been employed in health studies (Acevedo-Garcia et al. 2003; Collins and Williams 1999). Chiefly for this reason, we limited our measure of segregation to (un)evenness. Evenness measures the degree to which the proportion of a particular racial or ethnic group living in residential areas (e.g., census tracts) approximates that group’s relative percentage of an entire metropolitan area. It is measured using the dissimilarity index (D), which is interpreted as the proportion of the racial group of interest that would need to relocate to another census tract to achieve an even distribution throughout a metropolitan area. Although most health studies involving measurement of segregation are limited to dyadic comparisons, such as black/white segregation, we elected to incorporate the multigroup dissimilarity index (Dm), a version of the dissimilarity index generalized to capture concurrent segregation between multiple racial/ethnic groups (Iceland 2004; Sakoda 1981). The Dm has been developed to characterize segregation in the more typically multiethnic contemporary metropolis. We estimated multigroup segregation using the following formula:


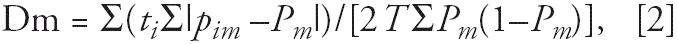


where *t_i_* is the number of residents in tract *i*, *p_im_* is the proportion of people in subgroup *m* in census tract *i*, *T* is the total number of residents in the metropolitan area, and *P_m_* is the proportion of people in subgroup *m* in the metropolitan area. The denominator sums the maximum segregation possible given the relative proportion of each racial/ethnic group in the metropolitan area. In sum, the numerator of the Dm is the minimum number of people who would need to move from one neighborhood to another so that the distribution of each racial/ethnic group in every neighborhood matches that of the metropolis as a whole. The denominator is the minimum number of people who would need to move to achieve this goal, starting from a context of complete segregation. Thus, the index varies from a value of 0, meaning no segregation exists (i.e., all neighborhoods have exactly the same distribution of people by race/ethnicity), to 1, complete segregation (i.e., each neighborhood is populated by only one racial/ethnic group). Intermediate values indicate a continuous range of racial/ethnic stratification of neighborhoods within a metropolis. One final note is that Dm is not composition dependent; consequently, this measure can be used to compare a diverse array of metropolitan areas, and it is not affected by the relative proportion of the demographic groups being examined.

Because air toxics concentration estimates were available only for the continental United States, we restricted our investigation to metropolitan areas within the same geographic reach. These metropolitan areas, as defined by the Office of Management and Budget based on data from the 1990 U.S. Census, are aggregations of counties that may (and often do) cross state boundaries. They are intended to describe an area dominated by a central city (with a population of at least 50,000) and surrounded by communities linked by housing and employment patterns (U.S. Bureau of the Census 1994). Because the HAP concentration data are available at the census tract level (1990 tract definitions), we used 1990 census tracts as a proxy for “neighborhood.” These areas are defined in advance of the decennial censuses and are nonoverlapping, mutually exclusive divisions of territory. Census tracts are nested within county boundaries and are intended to describe areas that are roughly comparable in population size (most tracts contain between 1,000 and 8,000 residents) and roughly consistent internally with respect to socioeconomic conditions. Some limitations of using census tracts as an approximation for neighborhoods have been described (Krieger et al. 2003). In addition, census tracts are the only construct approximating neighborhoods defined with a consistent methodology across all metropolitan areas of the United States.

We based our calculations on numbers of people in six exhaustive and nonoverlapping racial/ethnic groups as defined in the 1990 U.S. Census (U.S. Census Bureau 1991, 1993): Hispanics of any race, non-Hispanic whites, non-Hispanic blacks, Asians and Pacific Islanders, American Indians and Alaska Natives, and persons of “other” races. We recalculated these indices excluding persons of “other” races. Finding no substantive differences from our earlier calculations, we elected to retain this group in order to capture 100% of the population in each metropolitan area. We stratified the metropolitan areas into three segregation groups for further analysis: low to moderately segregated (Dm = 0.16–0.39), highly segregated (Dm = 0.40–0.60), and extremely segregated (Dm ≥ 0.60).

#### Regional grouping of states.

**Figure 1:**
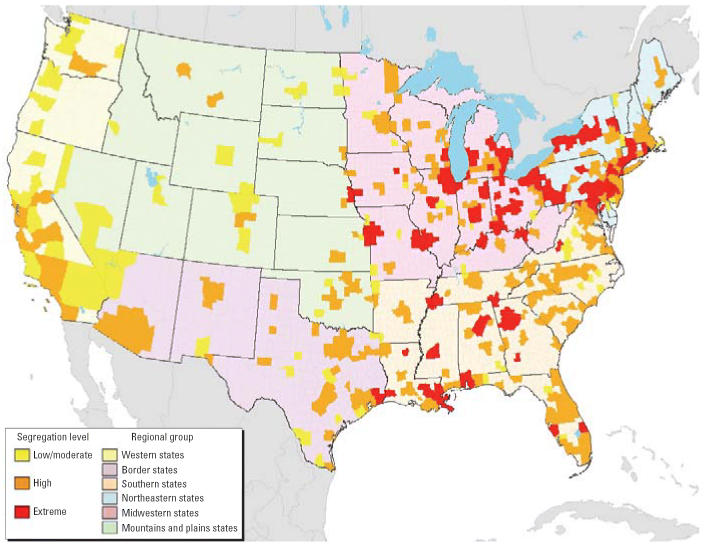
National map of multigroup racial/ethnic segregation in the United States (1990 Census; U.S. Census Bureau 1991, 1993).

Because previous research has documented regional variation in both the level of racial/ethnic segregation and its causes (Frey and Farley 1996), we developed six broad regional classifications of the continental United States to control for these differences (Figure 1): western states, the three states bordering the Pacific Ocean; border states, the three states sharing a border with Mexico (other than California); southern states, those that ceded to form the Confederate States of America during the Civil War (other than Texas); northeastern states, those north of the Mason-Dixon line and predominantly east of the Appalachian mountains (Pennsylvania, Maryland, the District of Columbia, and points northeast); mid-western states, from the western slopes of the Appalachians to the Mississippi River Valley (Ohio, West Virginia, and Kentucky west to Missouri, Iowa, and Minnesota); and mountains and plains states, those dominated by the central plains and Rocky Mountains (other than the border states).

#### Population density.

We estimated population density by dividing the number of residents in an area by the square kilometers of that area, as reported in the 1990 Census (U.S. Census Bureau 1991, 1993). Population density is often underestimated by this method because of the inclusion of large areas of uninhabited (and often uninhabitable) land area. To more accurately reflect the density of human habitation in each census tract, we dis-aggregated each tract into its constituent block groups (one to nine block groups per tract), estimated the population density for each block group, and then created a population-weighted sum of these population densities to estimate the average population density at which tract residents live.

#### Population size.

Researchers have noted that residential segregation of whites from blacks tends to be higher in metropolitan areas that are older and have larger populations and less recent growth in housing stock (Farley 1977). The influence of a city’s age on the level of black/white segregation is not independent of population size. Of these three measures, the population size of a metropolitan area has the clearest link to the volume and concentration of air pollution, even though this link is probably not independent of the local area population density described above. We categorized metropolitan areas into seven categories of population size defined by the Census Bureau, ranging from at least 50,000 to > 5 million (U.S. Census Bureau 1991, 1993).

#### Poverty and material deprivation.

To some degree, area level poverty may explain observed relationships between racial/ethnic segregation and estimated cancer risks associated with ambient air toxics exposures. Therefore, we examined poverty status as determined by 1990 U.S. Census household income and composition, in three categories: below the poverty level, above the poverty level but less than twice the poverty level, and at least twice the poverty level. The poverty level (which varies by household size and age composition) equaled $12,647 in 1989 for a family of two adults and two children (U.S. Bureau of the Census 2004). In addition to area-level poverty, we developed a census-tract measure of material deprivation by calculating a version of the Townsend index (Krieger et al. 2003; Townsend et al. 1988) adapted for U.S. census data by summing four *Z*-scores for the proportion of home owners, the proportion of car owners, the proportion of residents living in crowded conditions (at least one person per room), and the proportion of unemployed persons among workers.

#### Civic engagement.

Metropolitan areas characterized by racial/ethnic segregation may result in relative disenfranchisement of racial/ethnic minority groups. In a highly segregated metropolitan context, political influence and decision-making power are likely to be strati-fied across racial/ethnic lines and concentrated to serve the interests of racial majority communities (LaVeist 1992, 1993). This alignment of power could have implications for land-use decision making, transportation planning, and regulatory activities at a regional level in ways that affect ambient air quality in different neighborhoods (LaVeist 1992, 1993; Morello-Frosch 2002; Morello-Frosch et al. 2001; Pastor et al. 2001). We used a measure of voter turnout as a proxy for civic engagement, based on the number of votes cast in the 1996 presidential election (U.S. Bureau of the Census 1998) divided by the adult population in 1990. The finest geographic resolution for this data available across all metropolitan areas was at the county level.

### Statistical methods.

We calculated a descriptive statistic, population risk index (PRI), to assess potential environmental inequities across race/ethnicity, poverty level, and segregation categories. The PRI is a weighted average of the census-tract–level total cancer risk associated with ambient air toxics (Morello-Frosch et al. 2001; Perlin et al. 1995). The risk index is computed according to the following formula:


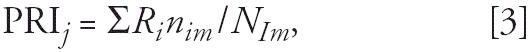


where *R_i_* equals the individual lifetime cancer risk estimate in census tract *i*, *n_im_* is the number of people in subpopulation *m* in census tract *i*, *I* is the set of all census tracts considered in the analysis (*I* = ∑*i*), and *N_Im_* is the total number of people in subpopulation *m* who reside in all tracts *I*. The population risk indices for different demographic groups can be compared with each other to graphically assess the extent to which environmental inequities may be occurring.

Because our exposure estimates are based on the ecologic unit of 1990 census tracts, we selected the Poisson regression technique to conduct multivariate modeling. To model relative exposure to carcinogenic air pollutants, we estimated rates of the expected number of lifetime cancer cases associated with modeled estimated ambient air toxics levels, by combining modeled concentration estimates with cancer potency information (IURs), and the population at risk in a given census tract. We divided the population of each tract into six categories based on race/ethnicity: Hispanics (of all races), non-Hispanic whites, non-Hispanic blacks, non-Hispanic Asians and Pacific Islanders, non-Hispanic American Indians and Alaska Natives, and non-Hispanics of other races. The outcome for our Poisson regression models was thus the expected number of cancer cases for members of each race/ethnic group in each census tract. A Poisson linear regression model with a robust standard error was used to estimate the average change in estimated cancer incidence associated with changes in segregation level and other covariates.

## Results

This analysis included 309 metropolitan areas encompassing 45,710 tracts and > 79% of the population of the United States, including 76% of non-Hispanic whites, 85% of non-Hispanic blacks, 91% of Hispanics (of any race), 87% of Asian/Pacific Islanders, and 53% of American Indians/Native Alaskans. The average individual lifetime cancer risk estimates for each metropolitan statistical area ranged across several orders of magnitude, with some of the highest risk estimates found in southern California and in the midwestern region (data not shown).

Table 1 presents the distribution of estimated cancer risk from air toxics in the U.S. census tracts. The average estimated cancer risk per million from all emissions sources combined was 631.9. This estimate declines significantly after removing diesel (115.5 per million; Table 2). Generally, cancer risk estimates exceeded the regulatory goal of one in a million by several orders of magnitude (Clean Air Act Amendments 1990). Among source contributions, mobile sources make the most significant contribution to estimated cancer risk (on average, 88.3% of total risk with diesel particulates included and 35.7% excluding diesel particulates). This is followed by area sources (7% including diesel particulates and 36% excluding diesel particulates) and then major point sources that contribute less on average to the overall cancer risk burden (1.3% including diesel particulates and 7% excluding diesel particulates).

Figure 1 maps patterns of racial segregation across the 309 metropolitan areas included in this analysis. The background colors indicate how we classified states into regional categories: western, border, southern, northeastern, mid-western, and mountains and plains states. The smaller, darker shapes are metropolitan areas. The map indicates that the northeastern, southern, and midwestern regions have some of the highest levels of multiethnic/racial segregation in the country, whereas the western and mountain and plains states tend to have lower levels of segregation. Table 3 displays the distribution of metropolitan areas, tracts, total population, and racial/ethnic groups by three segregation categories (moderate/low, highly, or extremely segregated). About 75% of metropolitan areas were either highly or extremely segregated (Dm ≥ 0.40), and nearly 40% of the census tracts included in this analysis were extremely segregated (Dm ≥ 0.60). Nationally, nearly 50% of non-Hispanic blacks, 37% of whites, more than 20% of Hispanics, and 24% of Asians live in extremely segregated metropolitan areas. These patterns vary significantly by geographic region, particularly in the northeastern and midwestern states, where segregation levels are highest.

**Figure 2:**
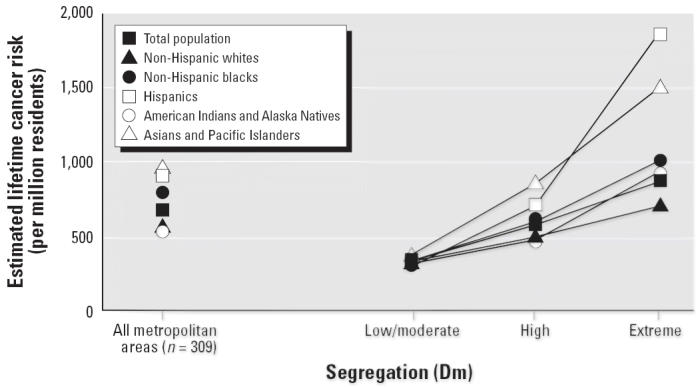
Estimated cancer risk associated with ambient air toxics by race/ethnicity and racial/residential segregation, continental U.S. metropolitan areas.

**Figure 3:**
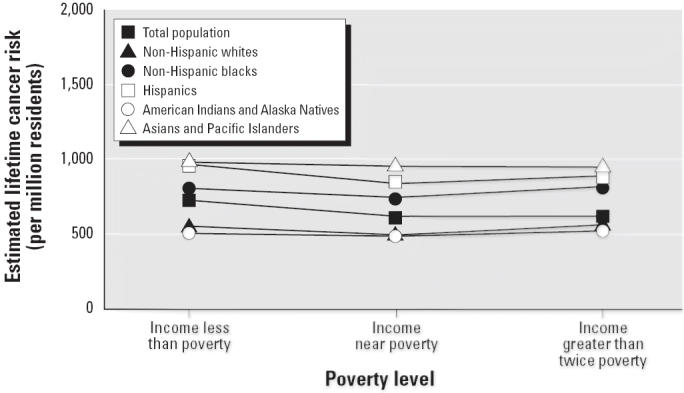
Estimated cancer risk associated with ambient air toxics by race/ethnicity and poverty status, continental U.S. metropolitan areas.

Figure 2 shows the racial/ethnic distribution of estimated cancer risk associated with air toxics across segregation categories. The *y*-axis shows a population-weighted individual excess cancer risk estimate for each racial/ethnic group and segregation category. Each line in the graph represents one of the five racial/ethnic groups, with one line representing the total population. The data points to the left are average cancer risk estimates for each racial/ethnic group for all segregation categories combined. The graph shows two patterns: that cancer risks across all metropolitan areas increase with increasing segregation levels for all racial/ethnic groups, and that overall, Hispanics and Asians, followed by African Americans, have some of the highest cancer risk burdens in metropolitan areas with higher segregation levels compared with the average risk across all groups and compared with whites and Native Americans. Figure 3 shows the racial breakdown of cancer risk burden by poverty level. Although there is a persistent racial/ethnic gap in cancer risk across all levels of poverty, there is no gradient that increases with rising area-level poverty, which suggests that the effect of segregation is independent of the impact of poverty on the exposure burdens across racial categories. The data were further examined to assess the racial/ethnic distribution of cancer risk across three segregation levels for each of the three area-level poverty categories. The same positive segregation gradient persisted for each racial group, regardless of poverty category (data not shown). This suggests that although segregation concentrates poverty (Massey and Fischer 2000; Massey et al. 1991), area-level poverty functions independently of segregation to affect estimated cancer risks associated with ambient pollutants. These distributional patterns were very similar when area and mobile source emissions were examined separately. For point-source emissions alone, the gradient across segregation categories was not observed (data not shown).

To examine these variables in a multivariate analysis, we assessed the relationship between segregation and estimated cancer risk, stratifying by race/ethnicity, and calculating risk ratios for each level of segregation, using low/moderate segregation as the referent group. Table 4 shows the unadjusted model without controlling for key area-level socioeconomic measures. This model shows a strong cancer risk gradient by segregation category for the total population [highly segregated: relative cancer risk (RCR) = 1.73; extremely segregated: RCR = 2.63] and indicates gradients for each racial/ethnic category with the strongest gradient observed for Hispanics (highly segregated: RCR = 2.44; extremely segregated: RCR = 6.40) and Asians (highly segregated: RCR = 2.25; extremely segregated: RCR = 3.90). Table 5 displays the adjusted model controlling for state regional grouping (six regions), metropolitan area population size, county-level voter turnout, tract-level poverty, tract-level material deprivation score (Townsend index), and tract-level population density. Results indicate that even after controlling for tract-level socioeconomic status (SES) measures, increasing segregation ampli-fies the cancer risks associated with ambient air toxics for all racial groups combined (highly segregated: RCR = 1.04; extremely segregated: RCR = 1.32). This effect of segregation is strongest for Hispanics (highly segregated: RCR = 1.09; extremely segregated: RCR = 1.74) but is also evident, albeit somewhat weaker, among whites, African Americans, and Asians. The models were also run for the source categories separately and showed strong gradients for mobile and area emission sources and nonsignificant effects for point sources (data not shown).

## Discussion

In this analysis we examined the relationship between estimated cancer risks from ambient air toxics, tract-level socioeconomic characteristics, and metropolitan-area racial segregation in the continental United States. Much of the average cancer risk is due to emissions from mobile sources, even when diesel particulates are removed from the analysis. We found a persistent relationship between increasing levels of racial/ethnic segregation and increased estimated cancer risk associated with ambient air toxics. Moreover, racial disparities in risk burdens widen with increasing levels of segregation. In examining race and tract-level poverty concurrently, we found a persistent disparity in population-weighted cancer risk among racial/ethnic groups across poverty levels. However, we observed no increasing gradient with increasing poverty, suggesting that segregation affects pollutant burdens in a manner independent of area-level poverty. Multivariate modeling controlling for tract-level SES variables showed that cancer risk burdens increased by increasing levels of segregation for all racial groups combined and that this positive relationship was most pronounced for Hispanics, whites, and blacks. Separate modeling by source category showed similar results for mobile and area emission sources, but not for point sources, where persistent segregation gradients for the total population and for each racial group were not observed.

Previous analyses of the U.S. EPA’s CEP and 1996 NATA data confirm the distribution of emissions source allocations for estimated cancer risk that are primarily driven by mobile sources (Apelberg et al. 2005; Morello-Frosch et al. 2000, 2001, 2002a, 2002b). Much of this difference in source contributions to estimated cancer risk for this study is driven by the overwhelming effect of diesel that is emitted by mobile sources. However, when diesel is removed from the analysis, mobile source emissions still account for 36% of estimated cancer risk. It is also possible that the difference in source contributions to estimated cancer risk is due to a lack of cancer potency information for those pollutants that tend to be released from stationary facilities (Morello-Frosch et al. 2000). The modeling results also confirm emerging evidence of racial disparities in exposure to air pollutants from mobile emission sources, including two studies in California examining traffic density and the demographic makeup of schools near major traffic corridors (Green et al. 2004; Gunier et al. 2003).

The segregation results in this study are consistent with those of one previous national study that examined the relationship between black/white residential segregation and ambient air toxics exposure in U.S. metropolitan areas using data from the U.S. EPA’s CEP (Lopez 2002). Results showed that increased black/white segregation was associated with wider disparities in potential air toxics exposure, after controlling for a series of area-level SES measures. We used a different methodologic approach in our study in terms of how we measured segregation, derived area-level SES measures, and developed our statistical models, yet the consistency of results between these two segregation studies is noteworthy. To our knowledge, our analysis is the only study to use a generalized multiethnic segregation measure for the evaluation of environmental health disparities.

Apelberg et al. (2005) recently conducted an analysis of racial and socioeconomic disparities in cancer risk associated with air toxics in Maryland using the NATA data and found substantial risk disparities for on-road, area, and nonroad sources by socioeconomic measures such as income, homeownership, education, and disparities in exposures from on-road and area sources by race (measured as percent black residents in a tract). Racial disparities in cancer risk were strongest at the lowest income levels (Apelberg et al. 2005). In our national study, we found persistent racial disparities across income categories, but this may be the result of differences in methodology in the estimation of race-based risks or in the demographic makeup of the different study areas. Moreover, we concentrated on segregation rather than on the proportion of specific racial groups in census tracts. Indeed, most environmental inequality studies use measures of racial composition or the existence of census tracts with a high proportion of specific minority groups to assess potential disparities. This measure of tract-level racial composition is often interpreted as a measure of the magnitude of segregation in a metropolitan area. However, racial composition may not always be a true reflection of segregation per se, because segregation is a contextual measure that depends on the relationship between racial groups in neighborhoods (e.g., census tracts) across a larger geographic area (e.g., a metropolitan area). Thus, whereas percent minority measures reflect the composition of a particular neighborhood, they do not assess whether a metropolitan area’s organization reflects broader patterns of racial inequality. Indeed, our results indicate that segregation, when operationalized as a measure of metropolitan area evenness, is associated with a higher average cancer risk overall and that it also amplifies disparities across racial groups, suggesting that this regional measure of inequality functions independently of neighborhood or tract-level SES measures.

There are some inherent limitations to this analysis, particularly related to the use of the NATA data. First, the characterization of health risks posed by air toxics focuses on additive cancer risks but says nothing about how some of these substances may interact synergistically with each other. Second, this analysis focuses on one route of potential exposure (inhalation through outdoor ambient exposures) and does not account for other exposure pathways through other media. Moreover, risk estimates do not take into account indoor and personal exposures to air toxics from other sources, such as consumer products, or the penetration of outdoor pollutants into indoor environments that can result in exposure levels that are significantly higher than estimated exposures from outdoor pollution sources. For example, ASPEN model estimates for volatile organic compounds used for NATA were generally lower than measured personal exposures and the estimated cancer risks (Payne-Sturges et al. 2004). Moreover, a comparison of the modeled air quality estimates with geographically limited ambient air monitoring data throughout the country found that the modeled estimates for the handful of pollutants examined by the NATA were typically lower than the measured ambient annual average concentrations (U.S. EPA 2005b). Another potential source of uncertainty arises from the comparison of 1996 risk estimates with racial and socioeconomic measures from the 1990 Census. We chose to use the 1990 Census to avoid having to arbitrarily exclude individuals who did not self-identify exclusively into one racial category. In terms of changes in pollution distributions, although emissions are likely to have changed during this period because of regulatory efforts, it is also likely that certain emissions—particularly the proliferation of mobile sources and the steady increase in the average number of vehicle miles driven in certain regions—could be counteracting previous gains from tougher emission standards from other sources (Apelberg et al. 2005).

## Conclusion

Although the literature on segregation and health has expanded significantly in recent years, studies that specifically address segregation in the context of environmental health disparities are in their infancy. Communities concerned about environmental inequities have encouraged scientists, policy makers, and the regulatory community to consider the junctures of socioeconomic inequality, environmental protection, and public health. This study suggests that disparities in exposures to cancer risks associated with ambient air toxics are affected by the degree of racial residential segregation, and that these exposures may have environmental health significance for populations across racial/ethnic lines. Furthermore, the observed increase in cancer risk in more segregated urban areas is not modified by area-level poverty. Future research, incorporating new and better models of exposure, should include segregation as a key factor in the analysis. Moreover, although most research has focused on the health consequences of black/white segregation in metropolitan areas, other minority groups may be similarly affected. Finally, examining segregation among metropolitan areas promotes a regional perspective for understanding the dynamics that shape environmental health disparities. The rationale for taking such a regional perspective is based on previous research that strongly suggests that it is more fruitful to assess potential drivers of environmental health disparities at the regional level because economic trends, transportation planning, and industrial clusters tend to be regional in nature, and zoning, siting, and urban planning decisions tend to be local (Maantay 2002; Morello-Frosch 2002; Morello-Frosch et al. 2001). Therefore, future work that examines how health inequities play out across metropolitan areas could have implications for the development of localized interventions and policy initiatives that ameliorate fundamental drivers of environmental inequities among diverse communities.

## Figures and Tables

**Table 1:** Distribution of estimated cancer risks in continental U.S. metropolitan areas, per million.

	Mean	5th percentile	Interquartile range	95th percentile
All sources	631.9	129.3	272.4–696.5	1619.1
Background	23.0	23.0	23.0–23.0	23.0
Point (major) sources	7.9	0.1	0.6–6.2	26.3
Area sources	43.3	5.4	13.3–50.9	135.6
Mobile sources	557.6	94.8	223.9–605.7	1465.8
On-road mobile sources	178.5	39.3	90.9–227.9	422.8
Nonroad mobile sources	379.2	48.7	122.1–368.4	1097.8

**Table 2:** Distribution of estimated cancer risks in continental U.S. metropolitan areas (excluding diesel particulate matter), per million.

	Mean	5th percentile	Interquartile range	95th percentile
All sources	115.5	37.7	61.0–137.9	277.0
Background	23.0	23.0	23.0–23.0	23.0
Point (major) sources	7.9	0.1	0.6–6.2	26.3
Area sources	43.3	5.4	13.3–50.9	135.6
Mobile sources	41.3	6.7	18.7–51.2	102.9
On-road mobile sources	25.4	4.4	12.3–33.3	61.2
Nonroad mobile sources	15.9	1.8	5.6–17.5	44.7

**Table 3:** Distribution of racial/ethnic groups by level of metropolitan area segregation.

		Segregation [Dm (%)]		
	Total (*n*)	Low and moderate 0.16–0.39	High 0.40–0.59	Extreme 0.60–0.82
Metropolitan areas	309	25	53	21
Census tracts	45,710	10	50	40
National	196,848,140	11	52	37
Hispanics of all races	20,386,166	13	66	21
Non-Hispanic whites	144,397,690	12	51	37
Non-Hispanic blacks	24,873,268	5	45	50
Non-Hispanic American Indians and Alaska Natives	894,954	21	60	19
Non-Hispanic Asians and Pacific Islanders	6,069,605	12	64	24
Western states	34,819,823	33	67	—
Hispanics of all races	7,756,347	20	80	—
Non-Hispanic whites	21,565,910	42	58	—
Non-Hispanic blacks	2,256,761	21	79	—
Non-Hispanic American Indians and Alaska Natives	233,259	50	50	—
Non-Hispanic Asians and Pacific Islanders	2,947,432	18	82	—
Southern states	39,028,191	5	71	24
Hispanics of all races	1,983,575	2	89	9
Non-Hispanic whites	28,404,970	5	72	23
Non-Hispanic blacks	7,995,229	5	63	32
Non-Hispanic American Indians and Alaska Natives	110,127	10	72	18
Non-Hispanic Asians and Pacific Islanders	514,659	5	74	20
Mountains and plains states	10,125,466	44	45	11
Hispanics of all races	685,376	51	43	5
Non-Hispanic whites	8,507,657	44	44	12
Non-Hispanic blacks	565,269	26	54	19
Non-Hispanic American Indians and Alaska Natives	174,238	26	71	3
Non-Hispanic Asians and Pacific Islanders	184,341	52	40	8
Border states	18,113,094	9	89	2
Hispanics of all races	4,620,933	14	85	0
Non-Hispanic whites	11,126,767	7	91	2
Non-Hispanic blacks	1,853,246	5	90	5
Non-Hispanic American Indians and Alaska Natives	135,802	4	95	1
Non-Hispanic Asians and Pacific Islanders	351,491	4	94	2
Midwestern states	43,620,713	3	26	72
Hispanics of all races	1,475,572	1	12	87
Non-Hispanic whites	35,856,980	3	29	68
Non-Hispanic blacks	5,463,371	1	10	90
Non-Hispanic American Indians and Alaska Natives	138,166	4	41	55
Non-Hispanic Asians and Pacific Islanders	656,826	3	25	72
Northeastern states	51,140,853	1	40	59
Hispanics of all races	3,864,361	0	29	70
Non-Hispanic whites	38,935,406	2	43	56
Non-Hispanic blacks	6,739,392	0	29	71
Non-Hispanic American Indians and Alaska Natives	103,362	3	35	63
Non-Hispanic Asians and Pacific Islanders	1,414,856	0	38	61

**Table 4:** Relative estimated lifetime cancer incidence associated with ambient air toxics [RCR (95% CI)], continental U.S. metropolitan areas.^a^

	Highly segregated	Extremely segregated
Total population	1.73 (1.69–1.77)	2.63 (2.57–2.70)
Non-Hispanic whites	1.55 (1.51–1.60)	2.19 (2.13–2.25)
Non-Hispanic blacks	1.90 (1.71–2.10)	3.18 (2.86–3.52)
Hispanics (all races)	2.44 (2.27–2.63)	6.40 (5.94–6.89)
Non-Hispanic American Indians and Alaska Natives	1.39 (1.05–1.85)	2.51 (1.85–3.39)
Non-Hispanic Asians and Pacific Islanders	2.25 (1.99–2.55)	3.90 (3.43–4.42)

CI, confidence interval. *R*^2^ = 5%.

a Unadjusted estimates.

**Table 5:** Relative estimated lifetime cancer incidence associated with ambient air toxics [RCR (95% CI)], continental U.S. metropolitan areas.^a^

	Highly segregated	Extremely segregated
Total population	1.04 (1.01–1.07)	1.32 (1.28–1.36)
Non-Hispanic whites	1.04 (1.01–1.08)	1.28 (1.24–1.33)
Non-Hispanic blacks	1.09 (0.98–1.21)	1.38 (1.24–1.53)
Hispanics (all races)	1.09 (1.01–1.17)	1.74 (1.61–1.88)
Non-Hispanic American Indians and Alaska Natives	1.02 (0.77–1.35)	1.21 (0.90–1.64)
Non-Hispanic Asians and Pacific Islanders	1.10 (0.97–1.24)	1.32 (1.16–1.51)

CI, confidence interval. *R*^2^ = 38%.

a Adjusted for state regional grouping; metropolitan area population size; county voter turnout; census-tract population density, poverty rate, and material deprivation.
